# MSTN^Del73C^ Mutation Modulates Glycerophospholipid Metabolism During Osteogenic Differentiation of Sheep BMSCs

**DOI:** 10.3390/cells15131136

**Published:** 2026-06-23

**Authors:** Luyu Wang, Yanhao Liu, Aowu Wu, Jingchao Cao, Yiping Zhao, Yibo Wang, Wenxi Ning, Zhengxing Lian, Kun Yu

**Affiliations:** Beijing Key Laboratory for Animal Genetic Improvement, National Engineering Laboratory for Animal Breeding, Key Laboratory of Animal Genetics and Breeding of the Ministry of Agriculture, College of Animal Science and Technology, China Agricultural University, Beijing 100193, China; zgwly2016@126.com (L.W.); yanhao.liu@cau.edu.cn (Y.L.); bs20243040464@cau.edu.cn (A.W.); s20233040741@cau.edu.cn (J.C.); zhaoyiping02@163.com (Y.Z.); zb20253040603@cau.edu.cn (Y.W.); yi1nwx@163.com (W.N.); lianzhx@cau.edu.cn (Z.L.)

**Keywords:** myostatin, bone marrow mesenchymal stem cells, osteogenic differentiation, metabolomics, lysophospholipid, gene editing

## Abstract

**Highlights:**

**What are the main findings?**

- Untargeted LC-MS/MS-based metabolomics revealed substantial metabolic remodeling during osteogenic differentiation of sheep BMSCs, particularly involving glycerophospholipid metabolism and multiple lysophospholipid subclasses.
- The MSTN^Del73C^ mutation was associated with distinct metabolic alterations during osteogenic differentiation, and targeted metabolomic analysis further validated the dynamic alteration pattern of LPC 18:2.

**What are the implications of the main findings?**

- These findings provide metabolomic insights into lipid metabolic remodeling during osteogenic differentiation in a large-animal model.
- This study expands current understanding of the potential association between MSTN mutation and metabolic regulation during osteogenic differentiation of sheep BMSCs.

**Abstract:**

Myostatin (MSTN) is a well-established negative regulator of skeletal muscle growth; however, its role in bone metabolism and osteogenic differentiation remains incompletely understood. In this study, untargeted and targeted metabolomic analyses were performed to investigate the metabolic effects of the MSTN^Del73C^ mutation during osteogenic differentiation of sheep bone marrow mesenchymal stem cells (BMSCs). Metabolomic profiles were analyzed in wild-type and MSTN^Del73C^ mutant BMSCs at 0, 7, and 14 days of osteogenic induction. During normal osteogenic differentiation, metabolites related to glycerophospholipid metabolism were repeatedly detected among significantly altered features, accompanied by marked increases in multiple lysophospholipid subclasses, including lysophosphatidylcholine (LPC), lysophosphatidylserine (LPS), and lysophosphatidylinositol (LPI). In contrast, MSTN^Del73C^ mutation was associated with significant reductions in several LPC and LPI species (*p* < 0.01 or *p* < 0.001), suggesting altered lipid metabolic profiles during differentiation. Targeted metabolomic validation further confirmed the altered abundance pattern of LPC 18:2. Collectively, these findings suggest that MSTN mutation is closely associated with metabolic remodeling during osteogenic differentiation and suggest potential involvement of glycerophospholipid-related metabolites involved in MSTN-related regulation of sheep BMSC osteogenesis.

## 1. Introduction

Metabolomics can comprehensively characterize the dynamic changes in small molecule metabolites and directly reflect the physiological state of cells, which makes it a powerful means to study the regulatory network under complex biological processes [1]. Bone marrow mesenchymal stem cells (BMSCs) are a kind of multipotent stem cell with the potential to differentiate into osteoblasts, chondrocytes and adipocytes, and are also a classic model for studying osteogenic differentiation and bone metabolism. Increasing evidence indicates that metabolic reprogramming is not merely a consequence of osteogenic differentiation, but also an active regulatory process governing stem cell fate commitment and osteoblast maturation [2,3]. During osteogenesis, coordinated alterations in lipid, nucleotide, and amino acid metabolism are closely associated with cell proliferation, extracellular matrix synthesis, and mineralization. Distinct metabolic programs are activated throughout different stages of osteogenic differentiation, reflecting dynamic requirements for bioenergetic transition, anabolic biosynthesis, and redox homeostasis that collectively support osteoblast maturation and mineralization [4,5,6].

Glycerophospholipids are not only essential structural components required for membrane biogenesis and matrix vesicle formation during osteogenesis, but also serve as bioactive signaling mediators involved in osteogenic pathways such as PI3K-AKT and Wnt/β-catenin signaling [7,8]. In particular, recent lipidomic studies have highlighted glycerophospholipid remodeling as a potentially important regulatory event during mesenchymal stem cell osteogenesis and senescence, possibly through its involvement in membrane dynamics, lipid signaling, and metabolic adaptation [9,10]. However, the metabolic profile of osteogenic differentiation in sheep BMSCs has not yet been fully characterized. Compared with rodent models, sheep exhibit more similarities to humans in terms of bone size, bone structure, and biomechanical properties, making them a valuable translational model for orthopedic and bone regeneration research [11,12].

Myostatin (MSTN) is a well-established negative regulator of skeletal muscle growth, and accumulating evidence suggests that it also participates in bone metabolism and osteogenic regulation [13,14]. In addition, MSTN deficiency has been associated with alterations in lipid, carbohydrate, and energy metabolism in livestock species [15,16,17]. Our previous metabolomic analysis of muscle satellite cells derived from MSTN^Del73C^ mutant sheep showed that during myogenic differentiation, nucleotide metabolism, unsaturated fatty acid biosynthesis, and the pentose phosphate pathway undergo significant reprogramming [18]. However, it is not yet fully clear whether MSTN mutations alter the metabolic program of osteogenic differentiation in BMSCs, particularly the remodeling of glycerophospholipid metabolism. Although metabolomic alterations during osteogenesis have been investigated in rodent and human stem cells [2,3,4,5,6], systematic characterization of lipid metabolic remodeling during osteogenic differentiation in large-animal models, particularly under MSTN-mutant conditions, remains largely unexplored.

To address the current knowledge gap regarding metabolic changes during osteogenic differentiation of sheep BMSCs and the potential association with MSTN mutation, the aim of this study was to characterize the metabolic alterations accompanying osteogenesis and to evaluate whether MSTN^Del73C^ mutation is associated with differences in metabolic profiles during this process. Untargeted LC-MS/MS metabolomics was employed to profile global metabolic features during osteogenic differentiation, followed by targeted validation of representative differential metabolites. By comparing metabolomic profiles under both non-induced and osteogenic conditions, we further assessed metabolic differences associated with MSTN^Del73C^ mutation. This study provides a metabolomic overview of osteogenic differentiation in sheep BMSCs and offers preliminary insights into metabolic alterations potentially associated with MSTN in bone metabolism.

## 2. Materials and Methods

### 2.1. Isolation, Culture, and Osteogenic Differentiation of BMSCs

BMSCs were isolated from the femurs and tibias of 6-month-old male Dorper sheep, including wild-type (WT) and heterozygous MSTN^Del73C^ mutant groups (n = 3 per genotype). MSTN^Del73C^ mutant sheep were created as previously described [19]. Briefly, Cas9 mRNA and MSTN-targeting sgRNA were microinjected into pronuclear-stage embryos, which were subsequently transferred into synchronized recipient ewes to obtain mutant offspring. All animal procedures were performed in accordance with the National Research Council guide for the care and use of laboratory animals and were approved by the animal care and use Committee of China Agricultural University (approval number AW02012202-1-3). Sheep BMSCs were isolated as follows: bone marrow was flushed from femur and tibia using DMEM/F-12 medium supplemented with 10% (*v*/*v*) fetal bovine serum (FBS) and 1% (*v*/*v*) penicillin streptomycin. The collected cells were cultured at 37 °C in a humidified incubator containing 5% CO_2_. After reaching 80–90% confluence, the cells were isolated and passaged using 0.25% trypsin EDTA. BMSCs at passages 2–5 were used for subsequent experiments. The identity of purified BMSCs was validated via multiple identification assays including immunofluorescence staining for MSC surface marker CD44, adipogenic differentiation induction and osteogenic differentiation induction prior to all functional experiments (Figures S1 and S2). For osteogenic differentiation, BMSCs were seeded into 6-well plates and cultured to approximately 80% confluence, and then induced with osteogenic medium consisting of low glucose DMEM supplemented with 10% FBS, 1% penicillin streptomycin, 50 μM ascorbic acid, 10 mM β-glycerophosphate, and 100 nM dexamethasone. After osteogenic induction, Alizarin Red S staining was used to preliminarily evaluate osteogenic mineralization.

### 2.2. Metabolomic Sample Preparation

Sheep BMSCs were cultured in 6-well plates and collected after non-induction (0 d), osteogenic induction for 7 or 14 days, respectively. Cells used for targeted and untargeted metabolomics were isolated from identical donor sheep but collected as separate cell batches in independent culture experiments. After removing the medium, the cells were washed twice with sterile PBS and then digested with 0.25% trypsin in an incubator at 37 °C, 5% CO_2_ for 2–3 min. After the cells were completely separated, an equal volume of serum-containing medium was added to terminate the digestion, and the cell suspension was collected into a sterile centrifuge tube. Cell numbers were determined using an automated cell counter, and all samples were adjusted to the same cell concentration. The cell suspension was centrifuged at 4 °C and 1000 rpm for 5 min, and the supernatant was discarded. Subsequently, 1 mL of pre-cooled methanol/acetonitrile/water solution (2:2:1, *v*/*v*/*v*) was added to each cell pellet, and then resuspended thoroughly. The cells were lysed by repeated freeze–thaw using liquid nitrogen for 3 times. The samples were then left at −20 °C for 1 h and centrifuged at 4 °C and 12,000 rpm for 15 min. The resulting supernatant was transferred to a fresh tube and dried using a vacuum centrifugal concentrator. For LC-MS/MS analysis, the dried extract was redissolved in 100 μL acetonitrile/aqueous solution (1:1, *v*/*v*), vortexed for 30 s, and subjected to ice bath ultrasonic treatment for 5 min. After centrifugation at 4 °C and 12,000 rpm for 15 min, the final supernatant was transferred to a sample bottle for subsequent metabolomic analysis. Quality control (QC) samples were generated by pooling equal volumes of all extracted samples.

### 2.3. Metabolomic Data Acquisition

Untargeted metabolomic profiling was performed using a SCIEX TripleTOF 6600+ quadrupole time-of-flight LC-MS/MS system (SCIEX, Framingham, MA, USA) coupled with an ExionLC AD ultra-high-performance liquid chromatography (UHPLC) platform. Chromatographic separation was achieved on an ACQUITY UPLC BEH C18 column (2.1 × 100 mm, 1.7 μm). The mobile phases consisted of water containing 0.1% formic acid (phase A) and acetonitrile (phase B). Gradient elution was conducted as follows: 0–2 min, 2% B; 2–13 min, 2–99% B; 13–16.5 min, 99% B; 16.5–16.6 min, 99–2% B; and 16.6–20 min, 2% B for column re-equilibration. The flow rate was maintained at 0.3 mL/min, the column temperature was set at 40 °C, and the injection volume was 5 μL. Mass spectrometric detection was performed using an electrospray ionization (ESI) source operated in both positive and negative ion modes. The mass scan range was set to *m*/*z* 60–1000. The ion spray voltage was 5500 V in positive ion mode and −4500 V in negative ion mode. Additional parameters were as follows: ion source temperature, 500 °C; declustering potential (DP), 80 V; collision energy (CE), 40 eV; curtain gas, 35 psi; and both nebulizer gas and auxiliary gas, 50 psi. Data acquisition was conducted in TOF-MS information-dependent acquisition (IDA)-MS/MS mode. Following each full scan cycle, up to 15 MS/MS spectra were automatically acquired according to ion intensity for subsequent metabolite identification and quantitative analysis.

### 2.4. Metabolomic Data Processing and Annotation

Raw LC-MS/MS data acquired in both positive and negative ionization modes were processed using the Dalian Dashuo MExplorer Ultimate high-resolution mass spectrometry analysis platform (V3.0.0.412). The workflow included chromatographic peak extraction, QC-based signal correction, feature filtering, and metabolite annotation. MS/MS spectra were subsequently matched with corresponding MS^1^ features in the peak table to improve metabolite identification accuracy. Following annotation, data from positive and negative ion modes were integrated, redundant features were removed, and the dataset was standardized through format conversion and normalization procedures. The resulting high-quality metabolite matrix was used for subsequent differential metabolite screening and multivariate statistical analyses.

### 2.5. Metabolomics Data Analysis

Preliminary filtering and quality control were applied to the raw metabolomic dataset prior to downstream analysis. Metabolite features were retained based on stringent criteria, including an annotation confidence score > 0.6 and a relative standard deviation (RSD) < 30%, to ensure high data reliability and reproducibility. Annotation confidence scores were calculated by integrating a metabolite standard reference database with large-scale MS/MS spectral datasets to achieve multi-level metabolite annotation. The filtered dataset was subsequently converted into CSV format and uploaded to MetaboAnalyst 6.0 for data normalization, log_2_ transformation of metabolite abundance, quality assessment, differential metabolite analysis, and pathway enrichment analysis. Statistical methods were selected according to the experimental design. Comparisons between two groups were performed using Student’s *t*-test, whereas one-way analysis of variance (ANOVA) followed by Tukey’s multiple-comparison test was applied for comparisons involving three or more groups. Multiple testing correction was performed using false discovery rate (FDR) adjustment. Multivariate statistical analyses, including principal component analysis (PCA), partial least squares discriminant analysis (PLS-DA), and orthogonal partial least squares discriminant analysis (OPLS-DA), were performed using MetaboAnalyst 6.0. Model performance was evaluated using R^2^, Q^2^, permutation testing and leave-one-out cross-validation (LOOCV) to assess robustness of multivariate models. Variable importance in projection (VIP) values derived from the OPLS-DA model were used to evaluate metabolite contribution to group discrimination, and metabolites with VIP > 1 were considered important differential variables. Differential metabolites identified from statistical analyses were further subjected to pathway enrichment and topology analyses in MetaboAnalyst 6.0 using the Ovis aries (sheep) KEGG pathway library to investigate their potential biological functions and associated metabolic pathways.

### 2.6. LC-MS/MS Targeted Quantitative Detection

A high-purity LPC 18:2 standard stock solution (5 mg/mL) was prepared in acetonitrile/methanol/water and serially diluted to generate a working solution (100 ng/mL) and a series of calibration standards ranging from 1 ng/mL to 2.5 μg/mL. All standard solutions were sealed and stored at 4 °C prior to analysis. Targeted quantification was performed using triple quadrupole LC-MS/MS following optimization of mass spectrometric parameters. The precursor ion and product ion were set at *m*/*z* 520.3 and 184.1, respectively, with a collision energy of 32 V and a declustering potential of 60 V. Chromatographic separation was achieved on a Phenomenex Synergi Hydro-RP C18 column (50 × 2 mm, 4 μm) using 0.1% formic acid in water and acetonitrile as the mobile phases. The flow rate was maintained at 0.5 mL/min, the column temperature at 40 °C, and the injection volume at 1 μL. Target metabolites were separated using a predefined gradient elution program. Calibration standards and biological samples were analyzed using the established LC-MS/MS method, and calibration curves were generated with SCIEX OS software (Version 2.0.1.48692). Instrument precision was evaluated by three consecutive injections, with relative standard deviation (RSD) values below 3%. LPC 18:2 concentrations in biological samples were subsequently calculated based on the corresponding mass spectrometric response values, and the analytical method was further validated prior to downstream analysis.

### 2.7. Statistical Analysis

All experiments were performed with at least three independent biological replicates. For technical replicates within each biological replicate, data were averaged prior to analysis. Data are presented as the mean ± standard error of the mean (SEM). Statistical analyses were conducted using GraphPad Prism 8 software (GraphPad Software, San Diego, CA, USA). Comparisons between two groups were performed using a two-tailed unpaired Student’s *t*-test. For comparisons involving three or more groups, one-way analysis of variance (ANOVA) followed by Tukey’s multiple-comparison test was applied. A *p* value < 0.05 was considered statistically significant. Statistical significance was indicated as follows: * *p* < 0.05, ** *p* < 0.01, and *** *p* < 0.001.

## 3. Results

### 3.1. Quality Control of Untargeted Metabolomics LC-MS/MS Data

**Figure 1:**
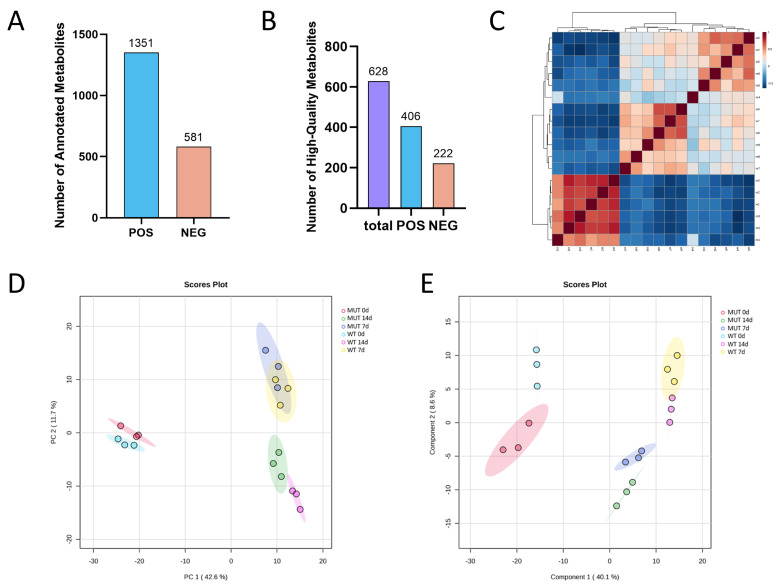
Quality assessment of untargeted LC-MS/MS metabolomic data. (**A**) Statistics of annotated metabolites detected in positive and negative ionization modes; (**B**) Distribution of high-confidence annotated metabolites after quality control filtering; (**C**) Correlation analysis among samples; (**D**) PCA score plot of samples from different groups; (**E**) Partial least squares-discriminant analysis (PLS-DA) score plot of samples from different groups.

Untargeted liquid chromatography-tandem mass spectrometry (LC-MS/MS) was employed to comprehensively profile metabolites in all experimental samples under both positive and negative ionization modes. Following peak picking, retention time alignment, baseline correction and preliminary database annotation, a total of 1351 metabolites were putatively annotated in positive mode and 581 in negative mode (Figure 1A). To ensure the robustness of downstream differential analysis, stringent multi-step quality control was implemented, including exclusion of low-quality features, QC-based filtering with a relative standard deviation (RSD) cutoff, and retention of high-confidence annotations with annotation confidence scores > 0.6. After sequential filtering, a refined high-quality non-redundant dataset comprising 406 positive-mode and 222 negative-mode metabolites was generated for subsequent multivariate statistical and differential metabolite analyses (Figure 1B).

Pearson correlation analysis was subsequently performed to evaluate intra-group consistency and overall data quality. Samples within the same group exhibited high correlation coefficients and tight co-clustering, while inter-group samples showed distinct correlation profiles (Figure 1C). These findings demonstrated excellent instrumental reproducibility, minimal technical variation and high intra-group biological consistency. Based on the QC-curated metabolite matrix, unsupervised principal component analysis (PCA) was conducted to explore global metabolic differences across groups. PCA revealed tight clustering of biological replicates and distinct separation between experimental groups, with no extreme outliers detected (Figure 1D). To explore potential group separation, a supervised partial least squares discriminant analysis (PLS-DA) model was constructed. The results showed a clear separation between groups, suggesting a good model fit and moderate predictive ability (R^2^ = 0.98685, Q^2^ = 0.5213), which was further supported by permutation testing (*p* = 0.025) (Figure 1E and Figure S3). Taken together, PCA and PLS-DA analyses indicated a consistent separation between experimental groups and suggested that the metabolomic data contained biologically relevant inter-group variation. These results provide a suitable basis for subsequent differential metabolite analysis, pathway enrichment, and functional interpretation.

### 3.2. Metabolic Differences During Osteogenic Differentiation of Sheep BMSCs

**Figure 2:**
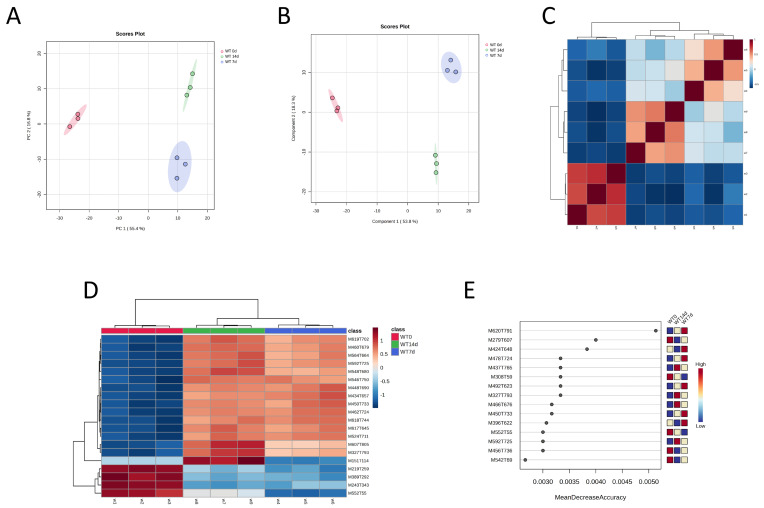
Global metabolomic profiling during osteogenic differentiation of sheep BMSCs. (**A**) PCA of metabolomic profiles among the three groups; (**B**) PLS-DA of metabolomic profiles among the three groups; (**C**) Correlation heatmap analysis of metabolites; (**D**) Hierarchical clustering heatmap of differential metabolites; (**E**) Identification of characteristic metabolites based on random forest analysis.

Successful osteogenic induction of sheep BMSCs was preliminarily evaluated by Alizarin Red S staining, which showed evident mineralized nodule formation after induction (Figure S1). To systematically characterize metabolic alterations associated with osteogenic differentiation of sheep BMSCs, multivariate statistical analyses and differential metabolite screening were performed using metabolomic profiles obtained from the non-induced group (WT-0d) and cells subjected to osteogenic induction for 7 days (WT-7d) and 14 days (WT-14d). PCA and PLS-DA analyses indicated a tendency toward separation among the three groups, accompanied by relatively tight intra-group clustering and partial inter-group discrimination (R^2^ = 0.99536, Q^2^ = 0.95826), suggesting possible group-associated metabolic variation during osteogenic differentiation (Figure 2A,B). Consistently, hierarchical clustering and correlation heatmap analyses revealed highly consistent metabolite expression patterns within groups and distinct metabolic profiles among groups (Figure 2C,D). In addition, a random forest model was applied to evaluate metabolite importance, and Mean Decrease Accuracy was used to quantify the contribution of individual metabolites to group discrimination, providing a reference for the identification of potential differential metabolic features (Figure 2E).

**Figure 3:**
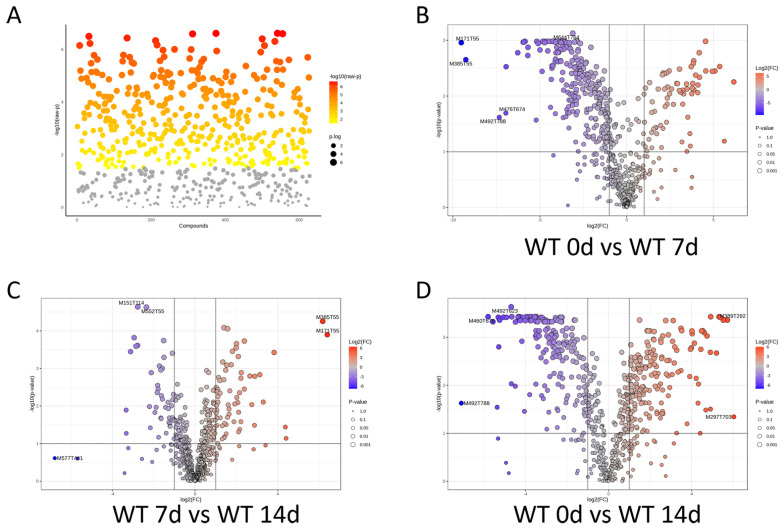
Comparative analysis of differential metabolites during osteogenic differentiation of sheep BMSCs. (**A**) Identification of differential metabolites based on one-way ANOVA; (**B**) Volcano plot of differential metabolites between WT-0d and WT-7d groups; (**C**) Volcano plot of differential metabolites between WT-7d and WT-14d groups; (**D**) Volcano plot of differential metabolites between WT-0d and WT-14d groups.

Differential significance analysis based on one-way ANOVA further confirmed extensive metabolic alterations among the three groups, providing a basis for subsequent pathway enrichment and mechanistic analyses (Figure 3A). To further characterize metabolic changes at different stages of osteogenic differentiation, pairwise comparisons were performed and visualized using volcano plots. In the WT-0d vs. WT-7d comparison, numerous metabolites showed significant alterations after 7 days of osteogenic induction. Among these, β-glycerophosphate (peak ID: M171T55), a component of the osteogenic induction medium, and LPC 24:4 (peak ID: M644T764) exhibited the most pronounced changes, reflecting both medium-related composition effects and cellular metabolic responses during early differentiation (Figure 3B). In the WT-7d vs. WT-14d comparison, β-glycerophosphate remained significantly elevated in WT-7d, whereas metabolites including xanthine (peak ID: M151T114) were markedly decreased, suggesting dynamic metabolic differences between intermediate and late stages of differentiation (Figure 3C). Furthermore, comparison between WT-0d and WT-14d revealed significantly higher levels of the tetrapeptide VAVT (peak ID: M389T292) and ricinoleic acid (peak ID: M297T703) in WT-0d, whereas LPC 16:2 (peak ID: M492T623) and LPE O-20:2 (peak ID: M492T788) showed lower abundance, suggesting extensive metabolic alterations throughout osteogenic differentiation (Figure 3D). Collectively, these pairwise comparisons provide visual and statistical evidence of metabolite abundance changes associated with BMSC osteogenic differentiation, while also accounting for the contribution of medium-derived components.

**Figure 4:**
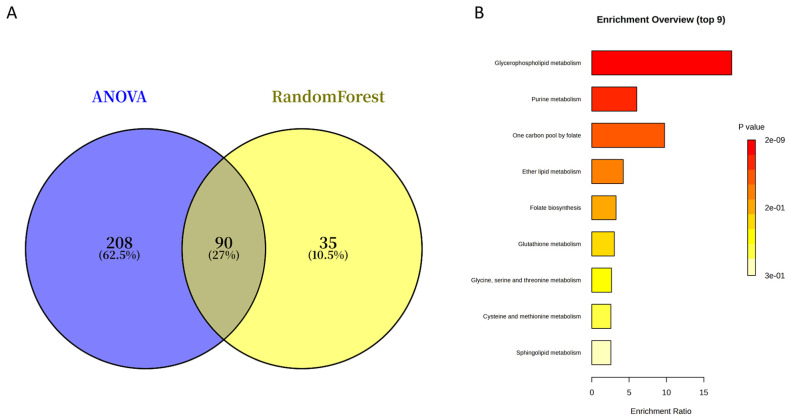
Screening and pathway enrichment analysis of differential metabolites during osteogenic differentiation of sheep BMSCs. (**A**) Venn diagram of differential metabolites identified by random forest analysis and one-way ANOVA; (**B**) Overview of metabolic pathway enrichment analysis for differential metabolites.

To improve the reliability and biological relevance of differential metabolite identification, conventional statistical analysis and machine learning-based feature selection were integrated in this study. Statistical significance for one-way ANOVA was defined as a false discovery rate (FDR) < 0.01 after Benjamini–Hochberg correction, whereas metabolites with a Mean Decrease Accuracy > 0.001 were considered important variables in the random forest model. Shared metabolites identified by both methods were visualized using a Venn diagram. ANOVA identified 298 differential metabolites, while random forest analysis screened 125 metabolites with high discriminatory importance. Among these, 90 metabolites were identified by both approaches and were subsequently selected for pathway enrichment analysis and further investigation (Figure 4A). Pathway enrichment analysis showed that these shared differential metabolites were mainly enriched in glycerophospholipid metabolism, purine metabolism, and folate-mediated one-carbon metabolism. Among them, glycerophospholipid metabolism exhibited the highest enrichment ratio and statistical significance (Figure 4B).

**Figure 5:**
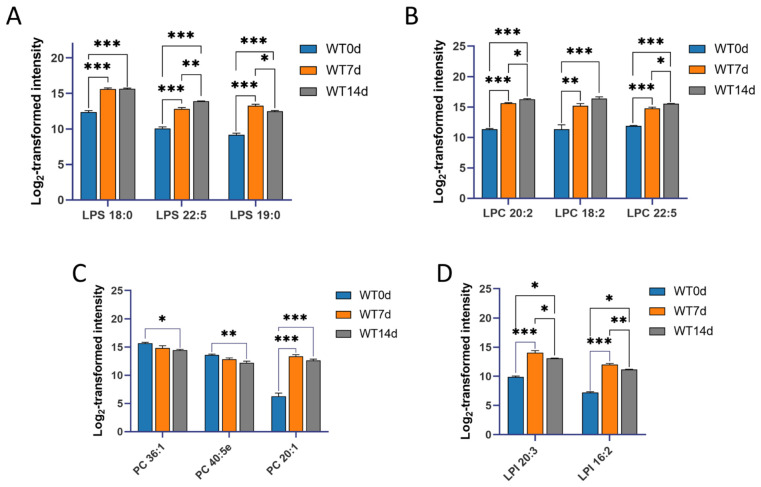
Abundance levels of key metabolites involved in glycerophospholipid metabolism during osteogenic differentiation of sheep BMSCs. (**A**) Abundance levels of three LPSs; (**B**) Abundance levels of three LPCs; (**C**) Abundance levels of three PCs; (**D**) Abundance levels of two LPIs. * *p* < 0.05, ** *p* < 0.01, *** *p* < 0.001.

To further investigate alterations in glycerophospholipid metabolism during osteogenic differentiation, the abundance patterns of key metabolites involved in this pathway were analyzed. Three lysophosphatidylserine (LPS) species, including LPS 18:0, LPS 22:5, and LPS 19:0, were significantly upregulated at both 7 and 14 days after osteogenic induction (*p* < 0.05) (Figure 5A). Among them, LPS 18:0 reached peak abundance at day 7 and remained elevated thereafter, whereas LPS 22:5 exhibited a gradual increase throughout differentiation. In contrast, LPS 19:0 peaked at day 7 and showed a slight decrease at day 14, although its abundance remained significantly higher than that in the WT-0d group. Similarly, three lysophosphatidylcholine (LPC) species, including LPC 20:2, LPC 18:2, and LPC 22:5, were significantly increased after osteogenic induction and displayed a sustained upward trend during differentiation (*p* < 0.05) (Figure 5B). Members of the phosphatidylcholine (PC) family exhibited distinct expression patterns: PC 20:1 was significantly upregulated and maintained at relatively high levels at both time points, whereas PC 36:1 and PC 40:5e were markedly downregulated, with PC 40:5e continuously decreasing during differentiation (*p* < 0.05) (Figure 5C). In addition, two lysophosphatidylinositol (LPI) species, LPI 20:3 and LPI 16:2, were significantly elevated after induction (*p* < 0.05). Their abundance peaked at day 7 and slightly declined by day 14 but remained markedly higher than that in WT-0d cells (Figure 5D). Collectively, these findings indicate that glycerophospholipid metabolism undergoes substantial alterations during the osteogenic differentiation of sheep BMSCs. Coordinated changes in multiple lysophospholipid subclasses (LPS, LPC, and LPI), together with differential regulation of PC species, suggest a close association between glycerophospholipid metabolism and the osteogenic differentiation process.

### 3.3. Metabolic Difference Analysis of BMSCs from MSTN^Del73C^ Mutant Sheep

**Figure 6:**
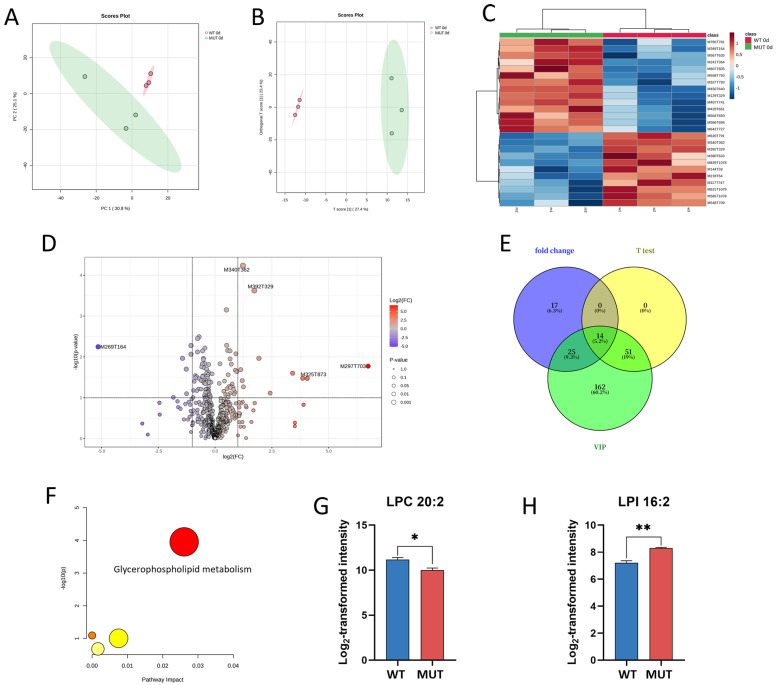
Metabolomic comparison between WT and MSTN^Del73C^ sheep BMSCs under non-induced conditions. (**A**) PCA of metabolite profiles between WT and MSTN^Del73C^ sheep BMSCs; (**B**) OPLS-DA of metabolite profiles between WT and MSTN^Del73C^ sheep BMSCs; (**C**) Hierarchical clustering heatmap of differential metabolites between WT and MSTN^Del73C^ sheep BMSCs; (**D**) Volcano plot of differential metabolites between WT and MSTN^Del73C^ sheep BMSCs; (**E**) Venn diagram of differential metabolites identified by VIP, fold change, and *t*-test analysis; (**F**) Bubble plot of pathway enrichment analysis for differential metabolites; (**G**) Abundance levels of LPC 20:2; (**H**) Abundance levels of LPI 16:2. * *p* < 0.05, ** *p* < 0.01.

To investigate metabolic alterations associated with the MSTN mutation, untargeted metabolomic profiling was performed in wild-type (WT-0d) and MSTN^Del73C^ mutant (MUT-0d) sheep BMSCs. PCA and OPLS-DA indicated a trend of separation between the two groups, accompanied by relatively tight clustering within groups and partial group discrimination. These findings suggest possible metabolic changes following MSTN editing (R^2^ = 1.000, Q^2^ = 0.778). (Figure 6A,B). Consistently, hierarchical clustering heatmap analysis further showed that WT-0d and MUT-0d samples formed two distinct clusters with highly consistent metabolic patterns within groups and distinct metabolic profiles between groups (Figure 6C). Volcano plot analysis identified numerous differential metabolites between WT-0d and MUT-0d cells. Among these, ricinoleic acid (peak ID: M297T703) exhibited significantly higher abundance in WT-0d, whereas adenosine (peak ID: M269T164) showed markedly lower abundance (Figure 6D). To improve the reliability of differential metabolite identification, three screening criteria, including fold change (|log_2_FC| > 1), Student’s *t*-test (*p* < 0.05), and variable importance in projection (VIP > 1), were applied simultaneously. Venn diagram analysis showed that 56, 65, and 252 metabolites were identified by the three methods, respectively, among which 14 metabolites satisfied all criteria (Figure 6E). These overlapping metabolites were subsequently selected for pathway enrichment analysis.

Pathway enrichment and topology analyses based on the 14 shared metabolites indicated that glycerophospholipid metabolism was the most significantly enriched pathway and exhibited the highest pathway impact value (Figure 6F). To further characterize alterations in this pathway, the abundance of representative metabolites was quantitatively analyzed. Compared with WT-0d cells, LPC 20:2 was significantly decreased in MUT-0d cells (*p* < 0.05), whereas LPI 16:2 was significantly increased (*p* < 0.01) (Figure 6G,H). These results indicate that MSTN mutation is associated with altered glycerophospholipid metabolism in sheep BMSCs. Collectively, the observed metabolic differences provide further evidence that MSTN mutation influences the basal metabolic state of BMSCs and suggest a potential association between glycerophospholipid metabolism and MSTN-related osteogenic regulation.

### 3.4. Metabolic Reprogramming Characteristics During Osteogenic Differentiation of BMSCs from MSTN^Del73C^ Mutant Sheep

**Figure 7:**
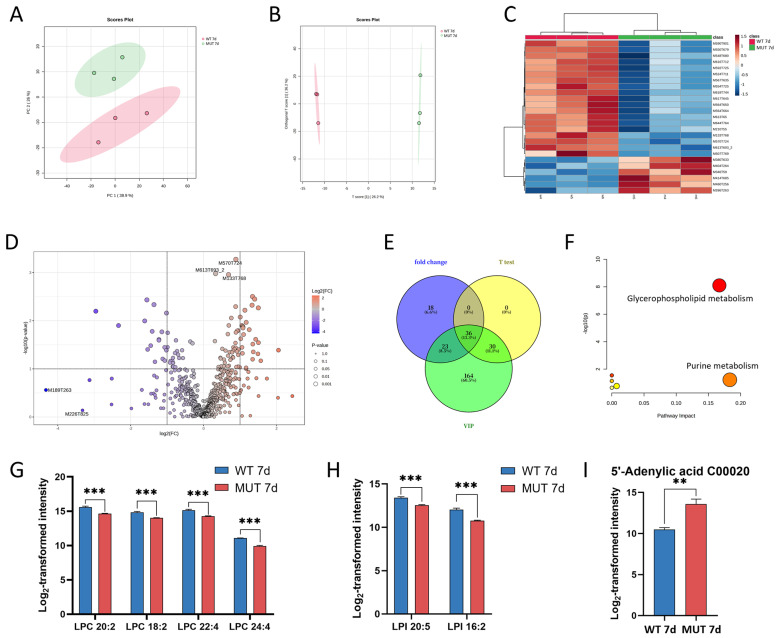
Metabolomic comparison between WT and MSTN^Del73C^ sheep BMSCs after 7 days of osteogenic induction. (**A**) PCA of metabolite profiles between WT and MSTN^Del73C^ sheep BMSCs after 7 days of osteogenic induction; (**B**) OPLS-DA of metabolite profiles between WT and MSTN^Del73C^ sheep BMSCs after 7 days of osteogenic induction; (**C**) Hierarchical clustering heatmap of differential metabolites between WT and MSTN^Del73C^ sheep BMSCs after 7 days of osteogenic induction; (**D**) Volcano plot of differential metabolites between WT and MSTN^Del73C^ sheep BMSCs after 7 days of osteogenic induction; (**E**) Venn diagram of differential metabolites identified by VIP, fold change, and *t*-test analysis; (**F**) Bubble plot of pathway enrichment analysis for differential metabolites; (**G**) Abundance levels of four LPCs; (**H**) Abundance levels of two LPIs; (**I**) Abundance levels of AMP. ** *p* < 0.01, *** *p* < 0.001.

To investigate metabolic alterations associated with the MSTN mutation during osteogenic differentiation, untargeted metabolomic profiling was performed in wild-type (WT-7d) and MSTN^Del73C^ mutant (MUT-7d) BMSCs following 7 days of osteogenic induction. PCA and OPLS-DA showed a tendency toward separation between WT-7d and MUT-7d cells, with relatively tight intra-group clustering and limited overlap, suggesting potential metabolic variation between the two groups (R^2^ = 0.997, Q^2^ = 0.898) (Figure 7A,B). Hierarchical clustering analysis further confirmed distinct metabolic expression patterns between the two groups (Figure 7C). Volcano plot analysis identified numerous differential metabolites, among which LPC 20:2 (peak ID: M570T724) was markedly decreased in MUT-7d cells (Figure 7D). To improve the reliability of differential metabolite identification, three screening criteria, including |log_2_FC| > 1, Student’s *t*-test (*p* < 0.05), and VIP > 1, were simultaneously applied. Venn diagram analysis showed that 77, 66, and 253 metabolites were identified by the three methods, respectively, among which 36 metabolites satisfied all screening criteria (Figure 7E). These shared metabolites were subsequently selected for pathway enrichment analysis.

Pathway enrichment and topology analyses indicated that glycerophospholipid metabolism was among the significantly enriched pathways (Figure 7F). Quantitative analysis further showed that four LPC species (LPC 20:2, LPC 18:2, LPC 22:4, and LPC 24:4) and two LPI species (LPI 20:5 and LPI 16:2) were significantly decreased in MUT-7d cells (*p* < 0.001) (Figure 7G,H). In contrast, 5′-adenylic acid (AMP), an important metabolite involved in purine metabolism, was significantly increased in MUT-7d cells (*p* < 0.01) (Figure 7I). These findings indicate that MSTN mutation is associated with substantial metabolic alterations during the early stage of osteogenic differentiation, particularly in glycerophospholipid and purine metabolism.

**Figure 8:**
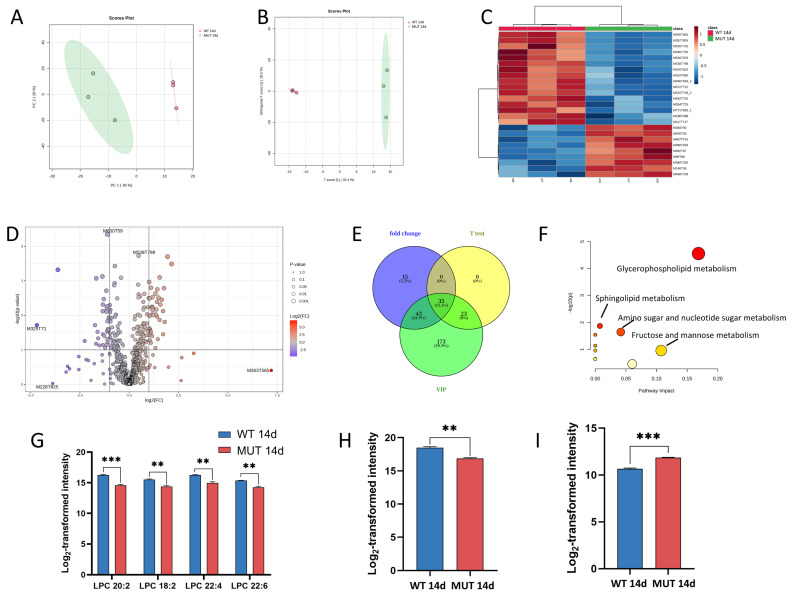
Metabolomic comparison between WT and MSTN^Del73C^ sheep BMSCs after 14 days of osteogenic induction. (**A**) PCA of metabolite profiles between WT and MSTN^Del73C^ sheep BMSCs after 14 days of osteogenic induction; (**B**) OPLS-DA of metabolite profiles between WT and MSTN^Del73C^ sheep BMSCs after 14 days of osteogenic induction; (**C**) Hierarchical clustering heatmap of differential metabolites between WT and MSTN^Del73C^ sheep BMSCs after 14 days of osteogenic induction; (**D**) Volcano plot of differential metabolites between WT and MSTN^Del73C^ sheep BMSCs after 14 days of osteogenic induction; (**E**) Venn diagram of differential metabolites identified by VIP, fold change, and *t*-test analysis; (**F**) Bubble plot of pathway enrichment analysis for differential metabolites. (**G**) Abundance levels of four LPCs; (**H**) Abundance levels of glycerophosphocholine; (**I**) Abundance levels of guanosine diphosphate mannose. ** *p* < 0.01, *** *p* < 0.001.

To further characterize metabolic alterations associated with MSTN mutation during the later stage of osteogenic differentiation, untargeted metabolomic analysis was subsequently performed in WT-14d and MUT-14d BMSCs. PCA and OPLS-DA showed a tendency toward separation between the two groups, together with relatively tight intra-group clustering, suggesting potential metabolic differences between the groups (R^2^ = 0.997, Q^2^ = 0.898) (Figure 8A,B). Heatmap analysis further confirmed distinct metabolic profiles between WT-14d and MUT-14d cells (Figure 8C). Volcano plot analysis identified multiple differential metabolites, including reduced LPC 19:0 (peak ID: M538T798) and increased uridine diphosphate N-acetylglucosamine (peak ID: M630T59) and uridine monophosphate (peak ID: M325T71) in WT-14d cells (Figure 8D). Using the same screening criteria, 35 shared differential metabolites were identified (Figure 8E). Pathway enrichment analysis showed that these metabolites were mainly enriched in glycerophospholipid metabolism, sphingolipid metabolism, and amino sugar and nucleotide sugar metabolism pathways (Figure 8F).

Within the glycerophospholipid metabolism pathway, four LPC species (LPC 20:2, LPC 18:2, LPC 22:4, and LPC 22:6) were significantly decreased in MUT-14d cells (*p* < 0.01), consistent with the metabolic alterations observed at day 7 (Figure 8G). In addition, glycerophosphocholine, an intermediate metabolite in glycerophospholipid metabolism, was also markedly reduced in MUT-14d cells (*p* < 0.01) (Figure 8H). In the amino sugar and nucleotide sugar metabolism pathway, guanosine diphosphate mannose was significantly increased in MUT-14d cells (*p* < 0.001) (Figure 8I). Collectively, these findings suggest that MSTN mutation is associated with persistent metabolic alterations during osteogenic differentiation of sheep BMSCs, particularly in glycerophospholipid metabolism.

### 3.5. Targeted Validation and Quantitative Analysis

To further validate the reliability of the untargeted metabolomics data, targeted validation was performed for quantitative verification. LPC is an important lysophospholipid involved in glycerophospholipid metabolism and membrane homeostasis, and previous studies have suggested its potential involvement in osteogenic differentiation. Based on its marked differential abundance in the untargeted metabolomic dataset, LPC 18:2 was selected as a representative metabolite for targeted validation.

**Figure 9:**
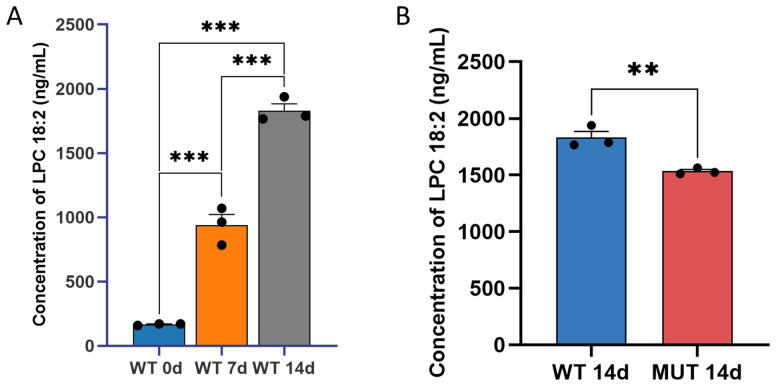
Targeted metabolomic validation of LPC 18:2. (**A**) Quantitative analysis of LPC 18:2 concentrations in WT sheep BMSCs at different stages of osteogenic differentiation; (**B**) Comparison of LPC 18:2 concentrations between WT and MSTN^Del73C^ sheep BMSCs after 14 days of osteogenic induction. ** *p* < 0.01, *** *p* < 0.001.

Untargeted metabolomic analysis initially identified significant differences in LPC 18:2 abundance among groups. Therefore, targeted quantitative analysis was subsequently performed to verify these alterations. The results showed that LPC 18:2 abundance gradually increased during osteogenic differentiation (*p* < 0.001) (Figure 9A), which was consistent with the trend observed in the untargeted metabolomic analysis (Figure 5B). In addition, comparison between WT-14d and MUT-14d cells demonstrated that LPC 18:2 levels were significantly reduced in MUT-14d cells (*p* < 0.01) (Figure 9B), consistent with the untargeted metabolomics results (Figure 8G). These findings support the reproducibility and reliability of the untargeted metabolomic data.

Collectively, targeted metabolomic validation confirmed the dynamic alteration of LPC 18:2 during osteogenic differentiation and its differential abundance following MSTN mutation. These results further suggest a potential association between LPC 18:2 and osteogenic differentiation of sheep BMSCs, while also supporting the involvement of glycerophospholipid metabolism in this process.

## 4. Discussion

This study shows a metabolomic framework for investigating metabolic alterations associated with the osteogenic differentiation of sheep BMSCs. By combining time-series metabolomic analysis with a comparison of wild-type and MSTN-mutant sheep cells, this study characterizes the dynamic metabolic changes during osteogenic differentiation and further assesses the metabolic disturbances associated with MSTN mutation. This study indicates that there is significant metabolic remodeling during osteogenic differentiation, with metabolites associated with glycerophospholipid metabolism were repeatedly identified among the significantly altered features across comparisons, and this pathway was enriched in pathway analysis. In addition, MSTN mutation was associated with persistent alterations in multiple lipid-related metabolic pathways during differentiation, indicating a possible link between MSTN and metabolic adaptation during osteogenesis.

During the osteogenic differentiation of sheep BMSCs, multivariate statistical analysis showed significant metabolic differences among the WT-0d, WT-7d, and WT-14d groups, indicating dynamic changes in metabolism at different stages of differentiation. In the enriched metabolic pathways, glycerophospholipid metabolism consistently shows high enrichment significance, indicating that it may be closely related to the progression of osteogenesis. Especially, the increase in various lysophospholipid subclasses, including LPS, LPC, and LPI, as well as the differential changes in several PCs, indicates that multiple metabolites related to glycerophospholipid metabolism exhibited significant changes during differentiation. These findings are generally consistent with previous studies showing that phospholipid remodeling is a common metabolic feature of mesenchymal stem cell osteogenesis. Bispo et al. (2022) reported that changes in lipid metabolism mainly occur in the early stages of osteogenic differentiation, with levels of phospholipids and unsaturated fatty acids increasing before reaching a relatively stable state [9]. Consistent with these observations, the results of this study also show that in the early stages of differentiation, several glycerophospholipid metabolites undergo significant changes. More recently, Bispo et al. (2025) further demonstrated that osteogenic differentiation is accompanied by dynamic turnover of phosphatidylcholine and phosphatidylethanolamine, which may contribute to membrane remodeling and mineralization-related metabolic adaptation [20]. Accumulating evidence indicates that phosphatidylcholine metabolism is crucial in osteoblast function, and a 2014 study revealed that blocking the CDP-choline pathway suppresses cell mineralization, proving that normal phosphatidylcholine metabolism facilitates osteoblast maturation [21]. Therefore, the differential regulation of PC species observed in the present study may reflect metabolic adaptation associated with membrane remodeling and osteogenic differentiation.

Among the altered glycerophospholipid metabolites identified in this study, LPC showed a consistent upward trend during osteogenic differentiation, suggesting its potential role in osteogenic-associated lipid remodeling. LPC is mainly produced through the hydrolysis of phosphatidylcholine mediated by phospholipase A2 (PLA2), although other metabolic pathways may also contribute to its formation [22]. Previous studies have further indicated a possible association between LPC and osteogenic differentiation. For example, Vickers et al. (2010) reported that LPC treatment promoted osteogenic-like phenotypic changes and mineralization-related gene expression in vascular smooth muscle cells [23]. Although this process was studied in the context of vascular calcification, the findings support that LPC-related lipid signaling may be involved in cell processes associated with calcification. In the present study, the coordinated increase in LPC together with alterations in LPS, LPI, and PC species suggests that glycerophospholipid remodeling may be associated with metabolic adaptation during osteogenic differentiation of sheep BMSCs.

In addition to glycerophospholipid metabolism, purine metabolism and folate-mediated one-carbon metabolism were also significantly enriched during osteogenic differentiation, suggesting increased metabolic demand for nucleotide synthesis and methyl donor metabolism during this process. Previous studies have shown that purine metabolism and purinergic signaling are closely associated with osteogenic differentiation of BMSCs. Extracellular ATP can be converted into adenosine through the sequential actions of CD39 and CD73, and adenosine subsequently regulates osteogenic-related signaling through purinergic receptors, particularly the A2B receptor pathway [24]. CD39/CD73 expression reduction and impaired adenosine signaling are also associated with bone loss in osteoporosis models [25], further supporting the importance of purine-related metabolic pathways in bone homeostasis. In addition, osteoblast mineralization is accompanied by increased phosphate utilization and elevated energy demands, which may further link purine metabolism to the osteogenic process [26]. In this study, the enrichment of purine metabolism may reflect metabolic adaptations related to nucleotide turnover and energy homeostasis during osteogenic differentiation. Folate-mediated one-carbon metabolism was also significantly enriched in our analysis. This pathway plays an important role in the generation of one-carbon units and S-adenosylmethionine (SAM), which are required for nucleotide biosynthesis and methylation-related cellular processes [27,28]. Previous studies have suggested that suppression of one-carbon metabolism impairs osteoblast mineralization and differentiation capacity [29]. Together, these findings suggest that metabolic pathways associated with lipid remodeling, nucleotide metabolism, and one-carbon metabolism may coordinately contribute to the metabolic adaptation required for osteogenic differentiation of sheep BMSCs.

It is noteworthy that MSTN mutations significantly alter the metabolic patterns observed during normal osteogenic differentiation. Even in the undifferentiated state (day 0), pronounced metabolic differences were observed between WT and MSTN^Del73C^ BMSCs, and these differences became even more pronounced after 7 and 14 days of osteogenic induction. Among the pathways identified by enrichment analysis, metabolites related to glycerophospholipid metabolism were consistently detected among the significantly altered features across different stages of differentiation. Specifically, certain LPC and LPI species that gradually increase during normal osteogenic differentiation were significantly lower in MSTN mutant cells, suggesting that MSTN mutation may be associated with changes in lipid metabolic remodeling during osteogenic differentiation. Previous studies have shown that dynamic phospholipid remodeling is important for osteogenic differentiation. The mutual conversion between LPC and PC is mainly regulated through the Lands cycle, and disturbances in this process are closely related to impaired osteogenic function. Tabe et al. (2024) reported that LPCAT2, a key enzyme involved in phospholipid remodeling, was upregulated during osteogenic differentiation, whereas LPCAT2 suppression impaired osteogenic marker expression and attenuated BMP/Smad signaling activation, suggesting that lysophospholipid remodeling contributes to osteoblast-like differentiation through regulation of osteogenic signaling pathways [30]. In this study, the sustained reduction in various LPC species in MSTN mutant cells may reflect changes in glycerophospholipid remodeling during osteogenic differentiation. Consistent with this interpretation, previous studies have suggested that LPC-associated signaling is involved in mineralization-related cellular responses [23]. Although the specific mechanisms between MSTN mutation and glycerophospholipid remodeling are not yet clear, our results indicate that the disruption of lysophospholipid homeostasis is closely associated with the metabolic phenotype changes observed in MSTN-mutant BMSCs.

In addition to glycerophospholipid metabolism, metabolites associated with amino sugar and nucleotide sugar metabolism were also altered in MSTN-mutant BMSCs during osteogenic differentiation. In particular, guanosine diphosphate mannose (GDP-mannose) was significantly increased in MUT-14d cells, suggesting potential disturbances in glycan-related metabolic processes. GDP-mannose serves as an important substrate for glycoprotein and N-glycan biosynthesis, and its abnormal accumulation may reflect altered glycosylation-associated metabolism during osteogenic differentiation. Previous studies have shown that glycosylation is closely associated with stem cell differentiation and extracellular matrix formation. Glycomics analyses demonstrated that mesenchymal stem cells undergo dynamic changes in N-glycan composition during osteogenic differentiation, including transitions from high-mannose glycans toward more complex glycan structures [31]. In addition, disruption of glycan synthesis has been reported to impair osteogenic differentiation and matrix mineralization [32]. More recently, Li et al. (2025) showed that altered N-glycosylation associated with ALG5 dysregulation affected osteogenic differentiation in osteoporotic BMSCs [33]. Combined with these previous findings, the altered GDP-mannose level observed in MSTN-mutant cells may indicate disturbed glycosylation-related metabolic adaptation during osteogenic differentiation. Together with the abnormalities identified in glycerophospholipid metabolism, these results suggest that MSTN mutation is associated with broad metabolic remodeling involving both lipid metabolism and glycan-associated pathways during osteogenic differentiation of sheep BMSCs.

Among the LPCs identified in this study, LPC 18:2 showed consistent and significant changes during osteogenic differentiation and was therefore selected for targeted metabolic validation. LPC 18:2 is an unsaturated lysophosphatidylcholine containing a linoleic acid chain and has been reported to participate in lipid signaling, inflammatory regulation, and cellular metabolic processes [34,35]. In the present study, targeted validation confirmed that LPC 18:2 abundance gradually increased during osteogenic differentiation, which was consistent with the trends observed in the untargeted metabolomic analysis. In addition, after 14 days of osteogenic induction, the levels of LPC 18:2 in MSTN-mutant BMSCs were significantly lower than those in wild-type cells, further supporting the results of untargeted metabolomics. The consistent change pattern observed in both untargeted and targeted analyses suggests that LPC 18:2 may be involved in metabolic remodeling during osteogenic differentiation. Given its dynamic changes during differentiation and its sensitivity to MSTN mutation, LPC 18:2 may represent a potential lipid metabolic marker associated with osteogenic differentiation of sheep BMSCs. More broadly, these findings further support the involvement of glycerophospholipid metabolism in the metabolic regulation of osteogenic differentiation.

Several limitations should be acknowledged in this study. First, the relatively small sample size, due to the limited availability of gene-edited large-animal-derived BMSCs, may affect the robustness of multivariate statistical analyses. Furthermore, the limited number of donor animals constrained the application of more complex statistical models for assessing inter-individual variability. Second, the number of significantly altered metabolites was relatively limited, which is partly attributed to the strict quality control and filtering criteria applied to ensure high-confidence metabolite annotation, as well as the inherent characteristics of untargeted metabolomics in primary cells, where biological variability and detection sensitivity may restrict metabolite coverage. Third, this study provides correlative metabolomic evidence only, and no functional validation experiments were performed. Future studies should incorporate targeted lipidomics to achieve a more comprehensive characterization of lipid metabolic remodeling, together with functional experiments to further elucidate the biological role of MSTN-associated metabolic alterations in osteogenic differentiation.

In summary, this study revealed the metabolic changes during the osteogenic differentiation of sheep BMSCs and found that MSTN mutation may be associated with the sustained metabolic remodeling in this process. Glycerophospholipid metabolism was among the pathways enriched in metabolite-based analyses, accompanied by dynamic changes in various lysophospholipid subclasses, including LPC, LPS, and LPI. In particular, the sustained reduction in several LPC species in MSTN-mutant BMSCs suggests a close association between altered glycerophospholipid metabolism and osteogenic differentiation. Furthermore, changes in pathways such as purine metabolism, one-carbon metabolism, as well as amino sugar and nucleotide sugar metabolism further indicate that osteogenic differentiation involves metabolic adaptations across multiple pathways. Collectively, these findings provide metabolomic evidence linking lipid metabolic remodeling to osteogenic differentiation in sheep BMSCs and expand the current understanding of metabolic regulation associated with MSTN mutation during bone development.

## 5. Conclusions

This study revealed dynamic metabolic remodeling during osteogenic differentiation of sheep BMSCs, particularly involving glycerophospholipid metabolism and lysophospholipid subclasses. Moreover, the MSTN^Del73C^ mutation was associated with distinct metabolic alterations during osteogenic differentiation. These findings provide metabolomic insights into osteogenic differentiation and metabolic remodeling associated with MSTN mutation in sheep BMSCs.

## Data Availability

All data supporting the findings of this study are available in the article or in the Supplementary Data Files or are available from the corresponding author upon request.

## Supplementary Materials

The following supporting information can be downloaded at: https://www.mdpi.com/article/10.3390/cells15131136/s1, Figure S1: Representative Alizarin Red S staining images of sheep BMSCs before and after osteogenic induction; Figure S2: Characterization of primary BMSCs; Figure S3: Cross-validation and permutation test of the PLS-DA model for six sample groups; Table S1: Metabolite Profile in Negative Ion Mode; Table S2: Metabolite Profile in Positive Ion Mode.

## Abbreviations

The following abbreviations are used in this manuscript:

MSTN	Myostatin
BMSCs	bone marrow mesenchymal stem cells
LPC	lysophosphatidylcholine
LPS	Lysophosphatidylserine
PC	Phosphatidylcholine
AMP	5′-adenylic acid
LPI	Lysophosphatidylinositol
LC-MS/MS	liquid chromatography-tandem mass spectrometry
RSD	relative standard deviation
PCA	principal component analysis
PLS-DA	partial least squares discriminant analysis
FDR	false discovery rate
OPLS-DA	orthogonal partial least squares discriminant analysis
SAM	S-adenosylmethionine
WT	wild-type
