# Supplementary material for: MSTNDel73C Mutation Modulates Glycerophospholipid Metabolism During Osteogenic Differentiation of Sheep BMSCs
Source: Cells. 2026 Jun 23;15(13):1136. doi: 10.3390/cells15131136 (PMC13359680; doi:10.3390/cells15131136)
Supplement: Supplementary file 1 [file cells-15-01136-s001.zip › Figure S1 Representative Alizarin Red S staining images of sheep BMSCs before and after osteogenic induction.pdf]

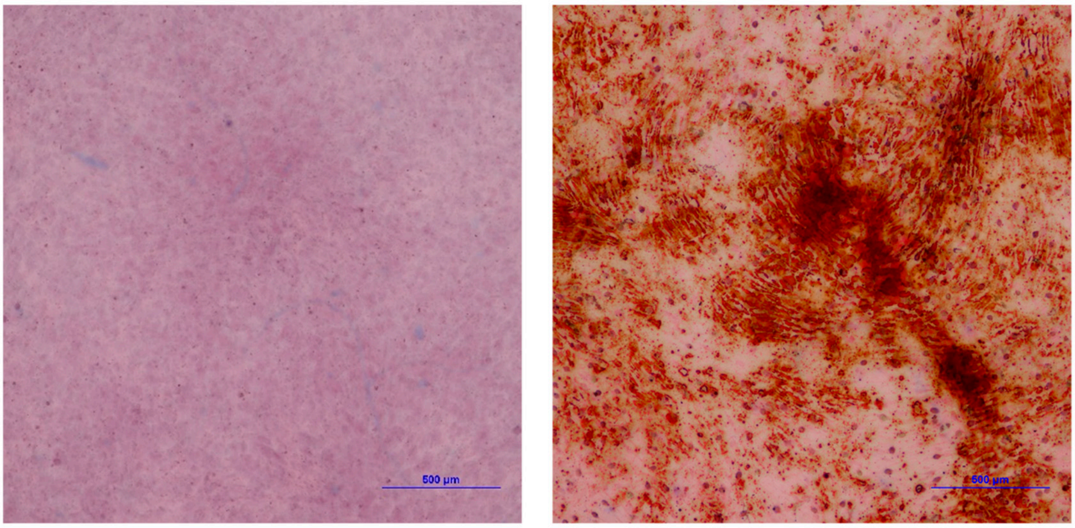

Before osteogenic induction After osteogenic induction

Figure S1. Representative Alizarin Red S staining images of sheep BMSCs before and after osteogenic induction. Minimal staining was observed before induction, whereas evident mineralized nodule formation was detected after osteogenic induction. Scale bar = 500 µm.
