# Supplementary material for: MSTNDel73C Mutation Modulates Glycerophospholipid Metabolism During Osteogenic Differentiation of Sheep BMSCs
Source: Cells. 2026 Jun 23;15(13):1136. doi: 10.3390/cells15131136 (PMC13359680; doi:10.3390/cells15131136)
Supplement: Supplementary file 1 [file cells-15-01136-s001.zip › Figure S2 Characterization of primary BMSCs.pdf]

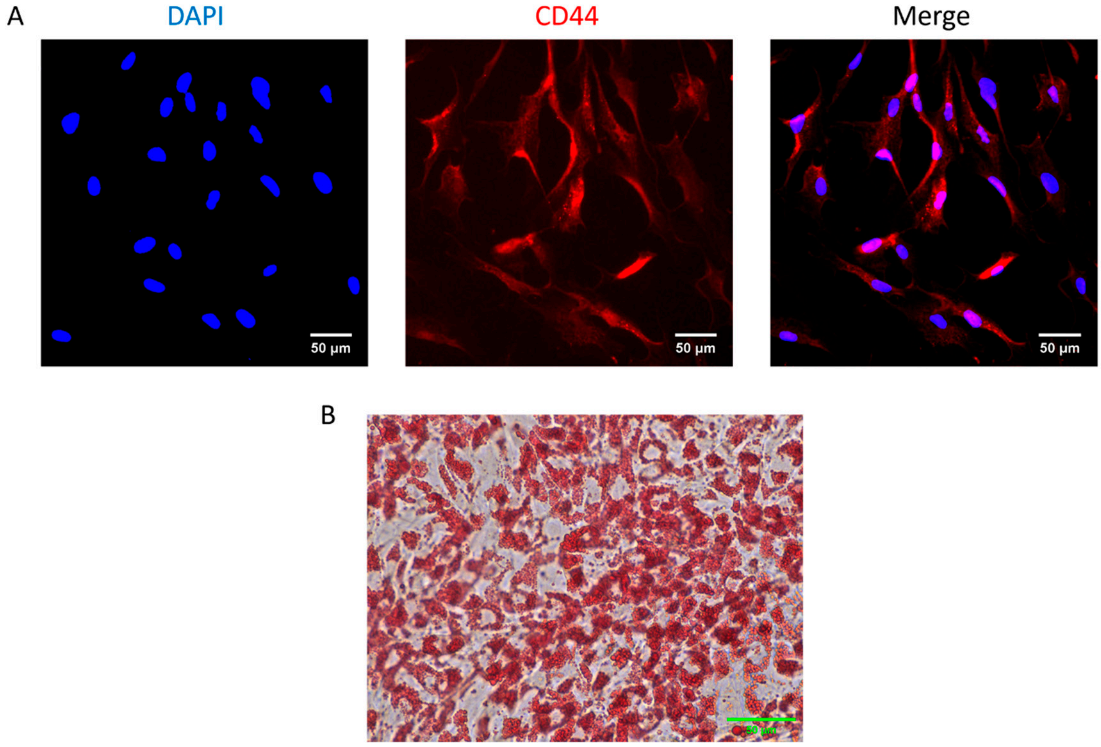

Figure S2. Characterization of primary BMSCs. (A) Representative immunofluorescence staining showing DAPI (blue, nuclei) and mesenchymal stem cell marker CD44 expression (red) in BMSCs. Right panel shows the merged image. (B) Oil Red O staining demonstrating adipogenic differentiation of BMSCs with intracellular lipid droplets (red). Scale bars = 50 µm.
