# Supplementary material for: MSTNDel73C Mutation Modulates Glycerophospholipid Metabolism During Osteogenic Differentiation of Sheep BMSCs
Source: Cells. 2026 Jun 23;15(13):1136. doi: 10.3390/cells15131136 (PMC13359680; doi:10.3390/cells15131136)
Supplement: Supplementary file 1 [file cells-15-01136-s001.zip › Figure S3 Cross-validation and permutation test of the PLS-DA model for six sample groups.pdf]

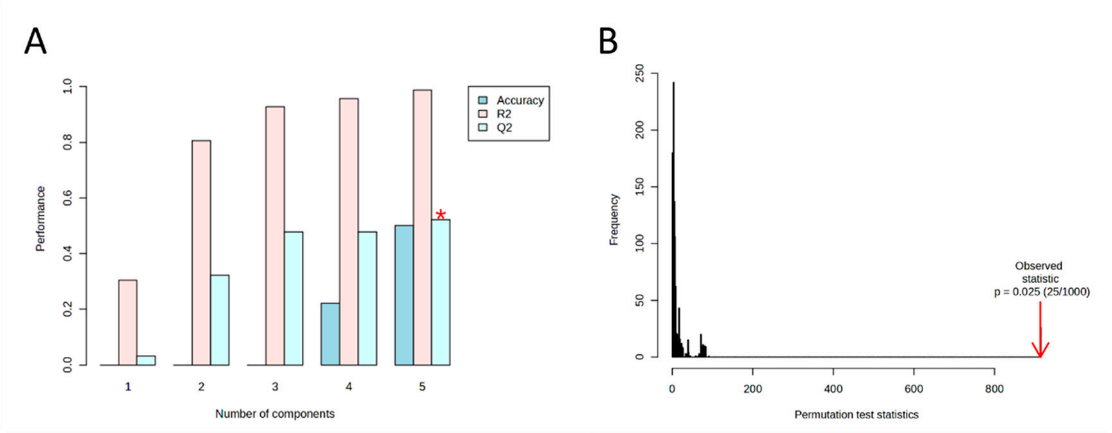

Figure S3. Cross-validation and permutation testing of the PLS-DA model for six sample groups. (A) Leave-one-out cross-validation (LOOCV) metrics, including accuracy, R^2, and Q^2, across different numbers of latent components. The red asterisk indicates the optimal model with five components. (B) Distribution of model statistics obtained from 1000 permutation tests. The red arrow indicates the statistic of the original PLS-DA model.
